# One Earth: The Equilibrium between the Human and the Bacterial Worlds †

**DOI:** 10.3390/ijms242015047

**Published:** 2023-10-10

**Authors:** Alicia Bravo, Ana Moreno-Blanco, Manuel Espinosa

**Affiliations:** Centro de Investigaciones Biológicas Margarita Salas, Consejo Superior de Investigaciones Científicas (CSIC), Ramiro de Maeztu 9, E-28040 Madrid, Spain

**Keywords:** antibiotic resistance, global regulators, horizontal gene transfer, mobilome, nichome, one earth, resistome

## Abstract

Misuse and abuse of antibiotics on humans, cattle, and crops have led to the selection of multi-resistant pathogenic bacteria, the most feared ‘superbugs’. Infections caused by superbugs are progressively difficult to treat, with a subsequent increase in lethality: the toll on human lives is predicted to reach 10 million by 2050. Here we review three concepts linked to the growing resistance to antibiotics, namely (i) the *Resistome*, which refers to the collection of bacterial genes that confer resistance to antibiotics, (ii) the *Mobilome*, which includes all the mobile genetic elements that participate in the spreading of antibiotic resistance among bacteria by horizontal gene transfer processes, and (iii) the *Nichome*, which refers to the set of genes that are expressed when bacteria try to colonize new niches. We also discuss the strategies that can be used to tackle bacterial infections and propose an *entente cordiale* with the bacterial world so that instead of war and destruction of the ‘fierce enemy’ we can achieve a peaceful coexistence (the *One Earth* concept) between the human and the bacterial worlds. This, in turn, will contribute to microbial biodiversity, which is crucial in a globally changing climate due to anthropogenic activities.

## 1. Introduction

The discovery of pathogenic bacteria and their potential to kill humans, animals, and plants triggered a long-term fight against the ‘deadly enemies’ that has been taking place over the years. There was a peak of justified optimism about a ‘total victory’ at the time of the discovery and first uses of antibiotics [1]. However, soon afterwards, bacteria carrying antibiotic resistance genes (ARGs) were isolated, and a race started (antibiotic-> resistance-> antibiotic, etc.) that has been worsening with time [2]. Thus, we have learned that the use of any antibiotic will lead to the selection of those bacterial clones and/or species that are resistant to them and, further, to the spread of these resistances to other bacteria. There is not a single process underlying resistance to a given antibiotic, on the contrary, resistance may be due to a variety of genetic, epigenetic, and biochemical mechanisms [3,4]. Throughout the years, the scientific and common language used has been war-like [5] and, even in our everyday commercials, we are advised to use cleansers that will ‘kill’ the fearful bacteria. Isolation of bacteria with multiple ARGs (the ‘superbugs’) has not contributed to reducing our fears, rather, on the contrary [6].

**Figure 1:**
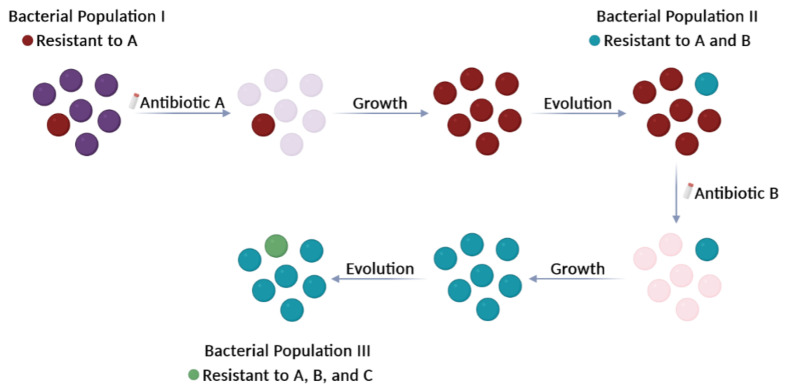
Selection of ‘superbugs’. Antibiotics are used to kill or significantly slow the growth of disease-causing bacteria. However, the use and abuse of antibiotics over time have led to the selection of bacteria that have accumulated multiple antibiotic resistances: mutations in chromosomal genes and/or ARGs acquired by HGT processes. These bacteria, known as ‘superbugs’, no longer respond to several types of antibiotics and continue to divide. Circles represent bacteria. Deep purple circles (Population I) indicate bacteria sensitive to antibiotic A. They will be killed (light purple circles) after treatment with antibiotic A. The colour of bacteria that are resistant to single or multiple antibiotics (Populations I, II, and III) is indicated in the Figure.

How have we arrived here? The use and abuse of antibiotics in medicine, production of foods, and livestock and crop protection have caused the selection of bacteria with resistance to those drugs (Figure 1). At present, antimicrobial resistance (AMR) is inevitable and irreversible because it is the result of a brutal selection of microorganisms that have been obliged to a fast and forced evolution during exposure to antimicrobials. The consequences are the following: (i) the present antimicrobials are losing effectiveness; (ii) the infections will be more difficult and expensive to treat, and (iii) epidemics will be hardly controlled. The World Health Organization (WHO) has predicted a human toll of ~10 million AMR-related deaths per year globally by 2050; see report on 17th November 2021 (https://www.who.int/news-room/fact-sheets/detail/antimicrobial-resistance; accessed on 31 July 2023) and requested urgent action on the subject. So far, the response of countries has been sloppy and insufficient. These dreary predictions have been supported by the report published in *The Lancet* by an international team [7]. Murray’s report, which is based on analyses of 204 countries over the past few years, calculated that up to five million people died in 2019 (before the official start of the COVID-19 pandemic) from illnesses in which drug-resistant bacteria played a role. This figure was increased by 1.2 million deaths directly due to the same cause. In the same year, AIDS and malaria caused 860,000 and 640,000 deaths, respectively. Most of the deaths caused by the superbugs were due to lower respiratory tract infections (mainly pneumonia) and sepsis from bloodstream infections. Currently, the most threatening infections caused by the deadliest superbugs include those caused by *Enterococcus faecium*, *Staphylococcus aureus*, *Klebsiella pneumoniae*, *Acinetobacter baumanii*, *Pseudomonas aeruginosa*, and *Enterobacter* spp., to which *Escherichia coli* has been added (the so-called ESKAPE-E group), most of them involved in nosocomial (hospital-acquired) infections. Murray’s report [7] also clarified that the prevention of infections and the appropriate use of existing antibiotics must be a policy to be followed by health agencies around the world. Discouragingly, most hospital treatment protocols include the routine use of antibiotics because of the risk of secondary bacterial pneumonia after viral infections. This is a fact that has been reported for the recent SARS-CoV-2 pandemic, which has resulted in a further selection of already drug-resistant pathogens [8]. Moreover, the Pan American Health Organization, the WHO’s Americas branch, has reported that the use of antimicrobials against SARS-CoV2 had resulted in a flood of drug-resistant infections, due to the prescription (and self-administration) of otherwise ineffective drugs against the virus [9]. The global dissemination of pathogenic bacteria poses another level of complexity to the surveillance and treatment of bacterial infections [10] and has led to the concept of ‘disaster microbiology’, defined as a ‘proposed field of study focused on the microbial impacts from severe storms and natural disasters’ (https://asm.org/Reports/Microbes-Climate-Change-Science,-People,-Impacts; accessed on 31 July 2023), and elaborated upon by Smith and Casadevall [11].

In nature, antibiotics do not reach the concentrations that are normally used in clinical treatments to kill the bacteria that are causing an infection. Further, the exposure of bacteria to the naturally present soil antibiotics does not take as long as in clinical treatments [12]. At present, we are faced with a lost cause if we do not change our views on the subject of AMR. As Louis Pasteur said (*circa* 1890): ‘Gentlemen, it is the microbes who will have the last word (Messieurs, c’est les microbes qui auront le dernier mot)’. The health crisis associated with the selection of superbugs should be conceived as an evolutionary and ecological dilemma. In addition, the human point of view of over-ruling competing organisms, other than bacteria (fungi, ‘bad’ weeds, and insects, to name a few), has important consequences on the global ecology and biodiversity of our planet, on climate change, and, as a consequence, on the evolution of the biosphere. A scientific warning has been issued on the importance of microorganisms in climate change [13].

Knowing these global evolutionary responses is of the essence if we are to participate in the race for climate change and biodiversity. Thus, to comprehend and tackle this crisis, we need to take an adequate perspective that must come about from evolutionary and ecological sources. The *One Earth* concept, developed by us [6,14] contemplates the entire biosphere as a whole entity where organisms must achieve an understanding to reach a global equilibrium while respecting the biodiversity of the populations [14]. Within the *One Earth* framework, the participation of the bacterial populations is crucial because of their role in biodiversity, abundance, and relevance to humans and their environments.

**Figure 2:**
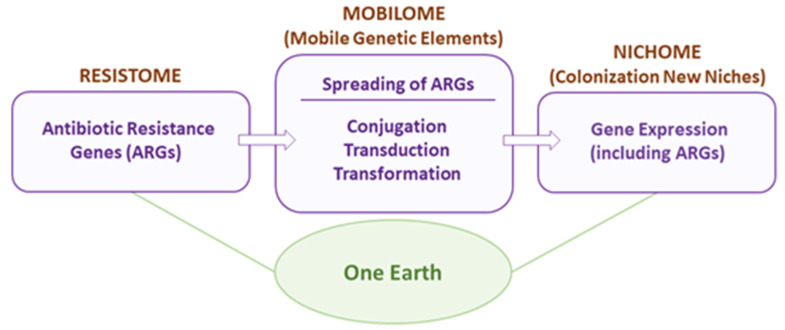
The *One Earth* concept. The relevance of the bacterial world to humans is implicit in the *One Earth* concept discussed in this review. Development of this concept requires detailed analyses of three notions related to the selection of AMR bacteria: the Resistome, the Mobilome, and the Nichome.

To modify the current fighting views [15], we conceived the concept of *One Earth* as a suitable approach to deal with infections due to AMR bacteria because it shows a way to reach an equilibrium between biodiversity (severely affected by the killing of these bacteria) and a reduction in bacterial virulence [14]. We shall review the different actors participating in the strife between the human and the bacterial worlds: the Resistome, the Mobilome, and the Nichome, as schematized in Figure 2. 

## 2. The Antibiotic Resistome

The collection of bacterial genes that can confer single or multiple AMR is termed **the Resistome** [3,16]. The resistome concept includes: (i) genes involved in resistance to a family of antibiotics (β-lactams, for instance); (ii) genes involved in resistance to multiple antibiotics; (iii) ARGs belonging to the microbiome of an individual; and (iv) ARGs of bacteria growing in specific niches, like hospitals, soils, or polluted waters [17]. A comprehensive antibiotic resistance database, termed CARD, has been updated and curated, and at present permits an in-depth search of the bacterial resistome, although the data are derived from cultivable and pathogenic bacteria [18]. An updated version of the previous ResFinder, termed ResFinderFG v2.0 (https://cge.food.dtu.dk/services/ResFinderFG/; accessed on 4 August 2023), which compiles 3913 ARGs identified by functional metagenomics from 50 curated datasets, has been recently released [19]. A search of the resistome in 349 lakes in Canada (from pristine to human-influenced places) revealed the existence of a natural aquatic resistome that might correspond to ARGs used in inter-bacterial communication and competition among different populations. Such a resistome was very much affected in areas where hospitals, human populations, and manure (used as fertilizers) increased the ARG diversity [20]. The most frequent source of AMR corresponded to acquired genes encoded by Mobile Genetic Elements (MGEs) that were shared among and transferred between bacterial species (see the Mobilome, below). Thus, the antibiotic resistome is an important hurdle that should be taken into account when new drugs are designed for therapeutic use [1]. Furthermore, the risk of failure in companies working on the discovery of new antibiotics has increased the need for private-public cooperation in drug discovery (https://wellcome.org/news/we-ignore-disaster-antibiotics-market-our-peril; accessed on 7 August 2023).

Novel technologies like deep-learning or atom-editing approaches are being used to find new, potentially useful antibiotics [21,22]. Interestingly, Artificial Intelligence (AI)-based approaches have discovered potentially interesting peptides derived from molecular de-extinction through paleo-proteome mining as a framework for antibacterial drug discovery [23]. A database to mine past and present human proteomes to search for potential bioactive encrypted peptides has recently been made available (https://gitlab.com/machine-biology-group-public/pancleave; accessed on 9 August 2023). 

**Figure 3:**
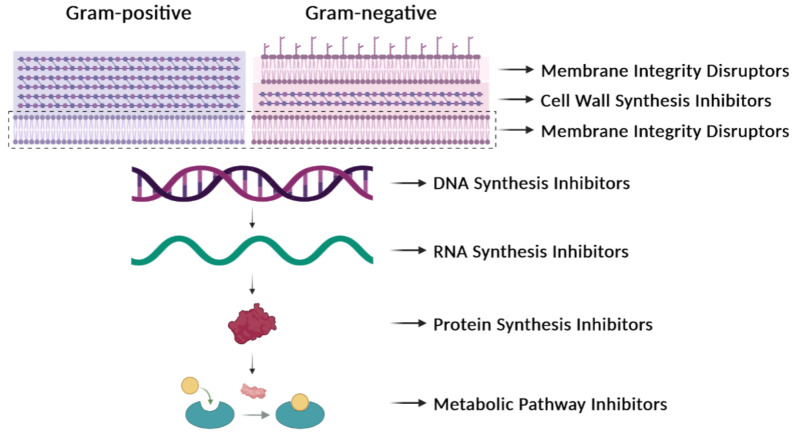
Antibiotic targets. Classification of antibiotics based on their mechanisms of action. They can affect the synthesis of the cell wall, the integrity of the membrane, the synthesis of nucleic acids (DNA or RNA), the synthesis of proteins, or the synthesis of particular metabolites. See Table 1 for additional information.

Our comprehension of infectious diseases caused by bacteria has increased very much but the rapid identification of new bacterial clones, novel mutations, and new variants of pathogens remains a challenge. Further, there are some obscure subjects that we do not fully understand, such as the factors that govern the individual immune response to pathogenic bacteria or how some of these pathogens can evade the immune response of the infected individuals. AMR has been known in bacteria for decades and the present status of a possible solution is still far from satisfactory. Nearly a quarter of a century has elapsed since the beginning of the 21st century, and we continue to be confronted with one of the most important public health issues, which is the increase in AMR over the years [24]. The proposed solutions to AMR involve the avoidance of the prescription of antibiotics to treat trivial infections, saving them for more serious ones. A reduction in the total consumption of antibiotics from 2011 to 2022 has been observed, and, despite this, AMR in bacteria has increased over these years, as reported by the European Centre for Disease Prevention and Control in March 2022 (https://www.ecdc.europa.eu/en/publications-data/antimicrobial-resistance-eueea-one-health-response; accessed on 31 July 2023). The accumulated knowledge on the mechanism of action of numerous antibiotics (Table 1, Figure 3) and the realization that several of them cannot be used because of widespread resistance make urgent the prevention of infections and the development of strategies to control them [1]. Furthermore, pangenome variability must be taken into consideration when developing new therapeutics that work at a species-wide level, because the variations in genomic organization and strain-specific variabilities may hinder conclusions on any given approach to develop new targets [4]. The O’Neill report (https://amr-review.org/; accessed on 7 August 2023) has been obscured by the serious outcomes of viral epidemics (Ebola, SARS-CoV-1) and, especially, the SARS–CoV-2 pandemics. And yet, the UK Health Office warns of a “hidden pandemic” of bacterial infections because one out of five people has antibiotic-resistant bacteria in their microbiome (https://www.bbc.com/news/health-59310099; accessed on 1 August 2023), probably due to an increase in the use of broad-spectrum drugs.

**Figure 4:**
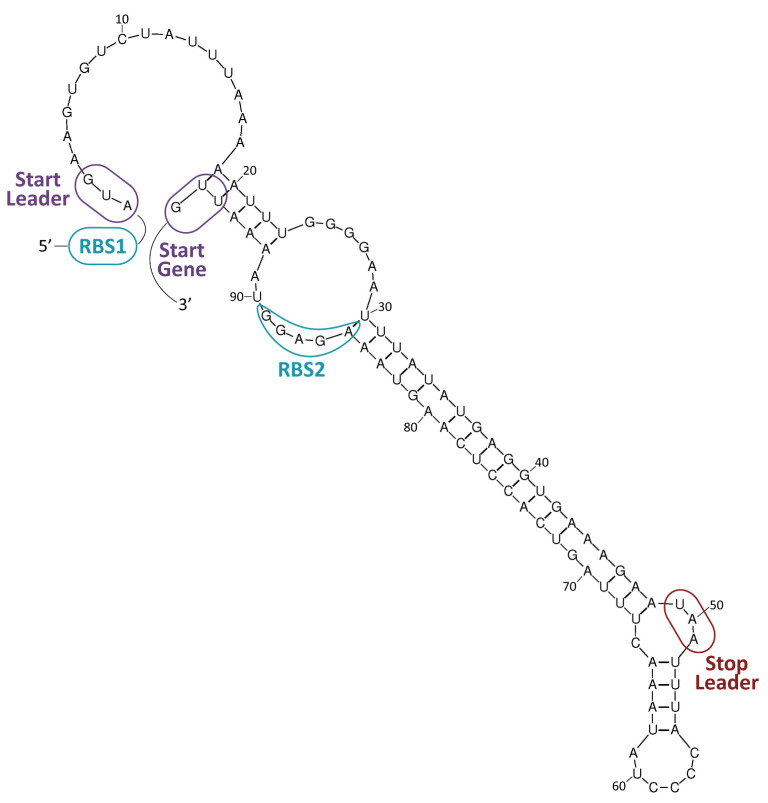
Resistance to tetracycline. The *tetK* gene of plasmid pT181 (GenBank accession number CP001783.1) encodes a tetracycline-resistance EP [28]. A potential secondary structure of its mRNA is shown. Formation of this structure would result in inhibition of translation. RBS1: proposed ribosome-binding site for the translation of a leader peptide from the AUG codon (start leader). RBS2: proposed ribosome-binding site for the translation of the *tetK* gene from the UUG codon (start gene). The UAA stop codon of the leader peptide is indicated. Expression of the *tetK* gene is inducible by tetracycline.

In general, antibiotics may be grouped by their mechanisms of action (Table 1, Figure 3), which affect: (i) cell-wall synthesis (β-lactams); (ii) protein synthesis (tetracyclines, phenicols, aminoglycosides); (iii) nucleic acid synthesis (rifamycins, fluoroquinolones); and (iv) membrane integrity (polymyxins) [25]. Resistance may be due to mutations in the bacterial chromosome or to ARGs carried by MGEs. Frequently, the resistance is the consequence of an enzymatic modification of the antibiotic molecules, like resistance to β-lactams or phenicols. There are several instances in which the resistances are due to Efflux Pumps (EPs), like those involved in resistance to tetracyclines. In several of these cases, a change in the cell morphology concomitant with the onset of antibiotic resistance has been observed [26]. EPs are transmembrane transporters able to pump the toxic molecules outside the bacterial cell and can be responsible for resistance to multiple antibiotics (reviewed by [27]). Constitutive expression of EP genes is a costly process, causing a heavy genetic burden to the bacteria carrying these genes. However, improved fitness can be achieved by organizing the EP genes in such a way that their mRNAs fold into secondary structures that make the translation inducible by the antibiotic. This is the case for several *tet* genes (tetracycline-resistance EP genes) [25,28]. Transcription of the *tet* genes results in the synthesis of an mRNA that folds into secondary structures that are more or less complex (Figure 4), as it has also been described for some *cat* (chloramphenicol-acetyl transferases) and *erm* (erythromycin-methylating enzymes) genes [29]. In all these genes, a single promoter directs the synthesis of an mRNA molecule that has two ribosome-binding sites (RBS1 and RBS2) (Figure 4) [30]. Translation from RBS1 leads to the synthesis of a short leader peptide (15–20 amino acid residues), whereas the resistance gene is translated from the downstream RBS2. Folding of the mRNA results in a secondary structure that occludes the RBS2 sequence, making it inaccessible to the ribosomes, thus inhibiting the translation of the gene. However, in the presence of small amounts of an antibiotic, drug-modified ribosomes translate the leader peptide from RBS1, which results in the refolding of the mRNA and exposure of the RBS2 sequence to other translating ribosomes [31]. Deletions that affect the formation of secondary structures lead to the constitutive expression of the *cat* gene [32].

The antibiotic resistome is dynamic and, at present, still expanding [16]. To tackle its study, the *One Health* philosophy contemplates the use of cutting-edge technologies, including Deep-Sequencing, AI, Big Data, and Bioinformatics, among them [33,34]. The latter two approaches have been recently used to perform an in-depth analysis of the resistome in samples from the Human Microbiome Project. It was found that nares had the highest load of ARGs (~5.4 genes per genome), followed by the oral cavity, whereas the gut exhibited the lowest abundance (~1.3 genes per genome). Interestingly, the transfer of common ARGs from humans to pristine environments seemed to be very low or nonexistent [35].

## 3. The Mobilome

The intra- and interspecific spread of antibiotic resistance among bacteria is increased by the existence of ARGs carried by MGEs. The mobile elements may represent up to 25% of the total prokaryotic DNA that is shared among bacteria. The ARGs can be mobilized intracellularly (from chromosome to plasmids and vice versa) or between different bacterial cells [36,37]. MGEs can be transferred among distantly related bacteria by Horizontal Gene Transfer (HGT) processes, constituting a profusion of circulating DNA termed **the Mobilome** [38]. It includes plasmids, Integrative and Conjugative Elements (ICEs), Integrative and Mobilizable Elements (IMEs), Insertion Sequences (ISs), and prophages and their satellites (Table 2). They all are involved in the transfer of AMR [39,40]. Nevertheless, the thin red line dividing the different MGEs is, at best, fuzzy because some ICEs and phages do replicate like plasmids, some phages and plasmids integrate into the host chromosome, and some satellites can integrate into phages [40,41].

### 3.1. Mobilization Mechanisms

The MGEs spread readily among their bacterial hosts, albeit with different efficiencies. Their dissemination can be achieved by different processes: (i) conjugation, a process that requires contact between donor and recipient cells; (ii) bacteriophage-mediated transduction; and (iii) transformation with free DNA, which occurs in naturally transformable bacteria [14]. The three processes may be equally efficient depending on the environment. If the bacterial cells are sessile and live in biofilms, where a matrix of free DNA is generated, natural genetic transformation may be the principal process [42,43,44]. When bacterial cells grow in dynamic environments, such as the mammalian gastrointestinal tract, conjugation could be the main HGT mechanism [45]. Furthermore, in bacteria containing prophages, lateral transduction can promote the transfer of large chromosomal regions at high frequencies [46]. Nevertheless, the situation may not be as linear as that because it has been shown that: (i) the presence of active prophages can seriously hinder the conjugation rates [47], and (ii) interactions between conjugative plasmids and ISs have significantly contributed to the horizontal transfer of AMR genes [48]. Further, it has been shown that the searching rate and the handling time are key parameters in plasmid conjugation. Moreover, both parameters are characteristic of the transfer machinery, rather than the entire plasmid genome [49]. At lower frequencies, yet importantly, two different plasmids can enter into a recipient cell by conjugation either simultaneously or by successive rounds of conjugation. These events would resemble an infective process and mathematical models have demonstrated the relevance of these processes in terms of the co-evolution of bacterial populations with their MGEs [50].

The classical **plasmid conjugation** process involves a specific protein (generically termed DNA relaxase), which initiates the transfer by binding to and cleaving at the plasmid origin of transfer, the *oriT*. The otherwise supercoiled plasmid DNA is relaxed and transferred to the recipient cell in a single-stranded DNA configuration [51]. There are, however, MGEs that ‘break the norm’ in the transfer process. There are, for instance, the *Streptomyces* plasmids, which follow a transfer path more akin to chromosomal segregation than to classical conjugation. The transfer of these plasmids is mediated by the TraB protein through a process that mechanistically resembles DNA translocation during the segregation of daughter molecules at cell division [52,53,54]. More extreme cases of mobilization are found in mycobacteria where the process termed Distributive Conjugative Transfer (DCT) implicates the transfer of chromosomal DNA segments from a donor cell to a recipient cell, independently of their location or genetic selection. The result of this process is the appearance of transconjugant cells harbouring patchwork genomes [55]. In addition, conjugative plasmids have evolved mechanisms to antagonize defense systems in the recipient cells [56].

**Bacteriophage-mediated transduction** has been known for decades to be a process to mobilize regions of the bacterial chromosomes [57] and small rolling-circle replicating plasmids [58,59]. More recently, the process termed lateral transduction has been reported, in which not only MGEs but also chromosomal genes are transferred at high frequencies [46]. These findings, in conjunction with more recent ones reported by the Penades lab [60,61], have posed the question of whether the bacterial chromosome could be conceived as an entire MGE, although differences can be conceived in terms of evolutionary selection [62].

**Natural transformation** is defined as the incorporation and integration of pieces of free DNA into the bacterial chromosome by recipient cells that have entered into a specific physiological state termed competence. So far, nearly 100 bacterial species have been shown to undergo this process for genetic variability. In some bacterial species, like *A. baumanii* (a most relevant member of the ESKAPE group), natural transformation seems to be the most relevant mechanism of HGT, posing the question of whether conjugative transfer is the main HGT process in bacteria [63]. In the case of polymyxins (a family of antibiotics that target the cell membrane), it has been speculated that treatment with these antibiotics could lead to the release of free DNA as well as potentiate the onset of the competent state [64]. The onset of competence is triggered by a specific sigma factor that turns on a set of operons (generically termed *com* genes) involved in the active incorporation of free DNA from the environment. The process, in several Gram-positive bacteria, is considered a bi-stable process in which two subpopulations of the bacterial strain differentiate: the non-competent fraction becomes the prey of the competent one, which would commit an act of fratricide or cannibalism [65,66,67,68,69]. The outbreak of competence was regarded as a survival mechanism in which the competent bacterial population would uptake and integrate some beneficial genetic traits into their chromosome. This would allow the transformed bacteria to survive unfavourable changes in the environment [70]. Niches shared by different bacterial species lead to ‘social interactions’ where transformation-mediated HGT could play an important role as a source of genetic variability and niche adaptation [71,72]. However, different views on natural transformation propose that the evolutionary function of this process is to cure bacterial genomes of their infectious parasitic MGEs [73]. Chromosomal integration of MGEs (new genetic traits) in times of need and removal of them by natural transformation when they are not needed would ensure both a strong dynamic of the bacterial genome in the short term and its long-term stabilization [43]. Thus, natural processes of gain or loss of function may be taking place repeatedly in natural environments. 

### 3.2. Transfer of MGEs

A thorough comparative study has quantified the frequencies of transfer of different MGEs and has reached two interesting conclusions: (i) most of the MGEs participating in HGT belong to the IME category rather than to plasmids, and (ii) less than 1% of the transferred genes are ARGs. Genes conferring other adaptive advantages, like those involved in host invasion or colonization of new niches, were frequently detected [74]. It might be argued, however, that HGT can be detected more easily if the gene is integrated into the host chromosome (IMEs, ICEs) than if carried by a plasmid because the latter can be lost in the absence of selective pressure. Thus, many events of plasmid-mediated HGT can occur in nature but only those that spread and remain in the population could be detectable [75]. It could also be argued that only plasmids carrying ‘addiction modules’ (i.e., Toxin-Antitoxin modules) that counter-select plasmid-free cells would be the most probable to remain in the population [76]. Acquisition of an MGE from the environment usually results in a loss of fitness by the recipient cells (measured as a reduction in the growth rate). Thus, in the absence of selective pressure, MGE-free cells can overgrow those that have recently acquired the MGE [77,78,79], at least until domestication processes take place [80]. Although mechanisms to avoid the fitness cost do exist (like those provided by the Toxin-Antitoxin modules), other strategies to maintain the advantage provided by the newly acquired MGE could be envisaged, such as (i) reduction in the copy number of the MGE by selection of mutations in the replication control elements; (ii) integration into the host chromosome (and, perhaps becoming an ICE); (iii) reduction in size and thus in the genetic load (small plasmids, IMEs); (iv) rapid domestication [80]; and (v) combination of two or more of the above instances [81].

### 3.3. Inhibition of HGT Processes

Strategies designed to inhibit the transmission of AMR traits by hindering conjugation were proposed early on. A search for Conjugation Inhibitors (COINS) through testing several libraries of natural compounds permitted the selection of some candidates as *bona fide* lead compounds [82]. Additional strategies were published later, like the employment of phage M13 g3p protein [83], followed by many interesting proposals to develop new COINS using either natural processes (CRISPR-Cas systems, restriction/modification) or genetically developed processes, like intrabodies against relaxases, obstruction of conjugative pili, or the use of unsaturated fatty acids [84,85,86]. Further, effective inhibition of bacterial conjugative transfer in natural environments has been demonstrated, showing the applicability of particular COINS to effectively block conjugation [87].

Additional approaches to reduce the spread of ARGs have been discussed in-depth [6]. These include interference with the stability of the mobilome, since by the same token by which MGEs can be acquired, they can be lost. Thus, drugs and processes that activate the loss of MGEs would be an attractive path to explore [88]. Another approach contemplates the inhibition of specific systems employed by bacteria to secrete macromolecules, like the T4 Secretion System (T4SS) that participates in conjugation [89]. Interestingly, inhibitors of the proton-motive forces in pneumococcus (COM-blockers) could inhibit the natural competence development in this bacterium, opening new ways to explore inhibitors of HGT focused on natural genetic transformation rather than on conjugative transfer [90]. 

Alternatives to tackle bacterial infections include, at least, the following approaches: (i) employment of inhibitors of protein-protein interactions; (ii) use of antisense RNAs; (iii) adoption of phage therapies; (iv) usage of nanoparticles as delivery agents; (v) employment of biofilm inhibitors; (vi) engineering Toxin-Antitoxin systems to use the toxins as alternative drugs; (vii) engineering broad-host-range plasmids equipped with CRISPR-*cas9* genes to block entry of plasmid DNA; and (viii) targeting ubiquitous genes by the use of the interference system CRISPRi. All these ways could be considered valuable tools to co-participate in controlling the spread of AMR [6,88,91,92,93,94]. Furthermore, monoclonal antibody-based strategies are being explored to treat bacterial sepsis and septic shock [95].

We can derive at least three lessons when trying to control HGT [96]. First, we must understand how universal the HGT processes to design strategies are to deal with them. Second, if we focus only on COINS and related strategies, we are neglecting natural transformation and transduction. Third, seeking universal HGT inhibitors may not be a good idea because we will severely affect bacterial genetic diversity.

## 4. The Nichome

The bacterial genome has been divided into the ‘core’ and the ‘accessory’ genomes. Whereas the core genome may amount to 20–60% of the bacterial genome, depending upon the species, the accessory genome contains genes that are present in one or more, but not all, strains of the species. These accessory genes might be beneficial when bacteria colonize new niches [97]. The mobilome is widely distributed and shared among bacterial species within the accessory genes. Thus, the mobilome can be considered an entity that actively participates in the intra- and interspecific gene spread, leading to an asymmetry in the bacterial populations. The accessory genes carried by the mobilome contribute to the genetic diversity of the bacterial world, and these variations confer advantages when bacteria explore the colonization of new niches [6,98]. In natural environments, most bacteria live in spatially structured communities composed of several species, the so-called polymicrobial biofilms [99]. Under these conditions, the sessile cells within the biofilm may release ‘explorers’ that would search for novel niches where they can either strive and prosper or be eliminated by other competitors or, in the case of infections, by the host immune system [100]. In the case of success, cells would colonize the novel niche and would express sets of otherwise silent (or nearly so) genes, **the Nichome** [6]. Genes participating in the nichome may represent an ample amount of the accessory genes, and they are involved in the niche-specific adaptation of the colonizing bacteria [101]. 

When colonizing a new niche, bacteria must be able to sense and respond to the signals that the new niche provides. Thus, the identification of genes responding to these signals will provide precious information on how the whole nichome is regulated. Within the niche, intraspecific signals are released (the quorum sensors, QS) that allow communication between kindred cells, which harbour receptors that respond to the signals released by cells of the same bacterial species, allowing them to cooperate [102]. For instance, several acyl-homoserine lactone-based QS systems have been identified [103]. Most interestingly, it appears that cells that share a given niche can monitor the QS signals emitted by different bacterial species, thus snooping on their presence, and making them able to establish cross-talks that would lead to either competition or, in some cases, cooperation [104]. The spatial organization of the biofilms (their biogeography) would greatly depend on the niche and, in the case of bacteria infecting humans, on (i) the infected organs; (ii) whether the infection is due to more than one bacterial species; and (iii) whether the infection is chronic [105]. The complexity of the polymicrobial nichome can be increased when some of the colonizing bacteria acquire MGEs from other species. In these cases, in addition to the genes expressed by the MGE, differential expression of chromosomal genes can occur, resulting in an alteration of the nichome genes that might be considered nonadaptive [106]. Acquisition of plasmids, for instance, has been shown to alter several bacterial traits that would help bacterial survival during (i) colonization; (ii) biofilm formation; (iii) virulence; and (iv) enhanced competition [107]. These changes in the gene expression patterns would result in an evolutionary advantage for those members who have acquired the MGEs. However, up to now we only possess preliminary information that points to a far more complex situation than previously envisaged. 

Bacterial adaptation to new niches requires coordination in the expression of numerous genes. This partially relies on regulatory proteins that activate and/or repress the transcription of various genes in response to environmental stimuli (global transcriptional regulators). Among them, (i) regulatory proteins that are associated with a transmembrane sensor histidine kinase, the so-called two-component signal transduction systems (TCSs); (ii) nucleoid-associated proteins (NAPs) that play an essential role not only in maintaining the DNA architecture but also in regulating gene expression; and (iii) regulatory proteins of the Mga/AtxA family, which has been defined as a new class of regulators containing phosphoenolpyruvate:carbohydrate **p**hospho**t**ransferase **s**ystem (PTS) regulation domains.

TCSs are widespread in bacteria but absent in animals and humans. Many studies illustrate their important roles in bacterial pathogenesis and antibiotic resistance [108,109,110]. The molecular mechanisms involved in the response to environmental changes by TCSs have been studied in detail [111]. In a prototypical TCS, the regulator generates a specific cellular response to the signal detected by the cognate histidine kinase. Most of the classified response regulators, but not all of them, contain a DNA-binding domain and are expected to function as transcriptional regulators. Generally, phosphorylation of this class of regulators by the histidine kinase increases the affinity of the regulator for specific DNA motifs, usually direct or inverted repeats. Thus, the activated regulator binds to its target promoters and alters (activates or represses) gene expression. Proteins that can modulate the activity of particular TCSs by interacting with either the histidine kinase or the response regulator have been identified [112,113,114]. In some cases, such proteins establish connections between TCSs and enable one system to respond to the signal detected by a different system [115]. Other strategies to regulate the activity of TCSs have been reported. For example, a recent study performed in *Bacillus anthracis* showed that an RNA-binding protein, named KrrA, controls post-transcriptionally the activity of the HitRS TCS by modulating the stability of the *hitRS* mRNA. Such a TCS responds to cell envelope damage [116]. Additionally, it has been shown that KrrA influences the expression of over 150 genes, including genes involved in genetic competence, transport, and metabolism [116].

NAPs are architectural proteins of the bacterial chromosome. It has been described that some of them participate in AMR [117]. Most of the NAPs play key roles as global regulators of gene expression, as is the case of H-NS (histone-like nucleoid structuring protein) and HU from enterobacteria [118]. H-NS functions generally as a transcriptional repressor. It regulates a wide range of genes in response to changes in osmolarity, pH, or temperature [119,120,121]. In vitro, DNA binding experiments have shown that H-NS does not recognize particular DNA sequences, but it has a strong preference for intrinsically curved AT-rich DNA regions [122]. Moreover, structural studies have shown that the DNA-binding domain of Ler, a member of the H-NS protein family, does not participate in base-specific contacts but recognizes specific structural features in the DNA minor groove [123]. In solution, H-NS can form higher-order oligomers, which correlates with its ability to form nucleoprotein filaments, either linear filaments (binding of H-NS to one DNA duplex) or bridged filaments (binding of H-NS to two segments of DNA duplex). Both types of nucleoprotein filaments can suppress transcription by different mechanisms [120,121,124,125,126,127]. It has been shown that H-NS selectively silences the expression of AT-rich horizontally acquired genes, including pathogenicity islands. This silencing is crucial during the integration of foreign genes into the host genome to ensure that bacterial fitness is maintained [128,129]. Proteins that can enhance H-NS-mediated silencing have been identified (e.g., Hha and StpA) [130,131]. Furthermore, in *Shewanella oneidensis*, H-NS was shown to silence various foreign genes by a mechanism that relies on H-NS phosphorylation at warm temperatures [132]. Interestingly, bacteria have evolved strategies of counter-silencing, which ensure the expression of H-NS-repressed foreign genes in particular niches and under certain environmental conditions. Some of such strategies are based on proteins (counter-silencers) that allow transcription initiation and/or transcription elongation through repressive H-NS nucleoprotein filaments [133,134,135]. Also, a counter-silencing mechanism involving H-NS degradation has been reported [136].

As well as H-NS, the bacterial NAP termed HU plays a critical role in global gene regulation. In *E. coli*, the HU regulon includes genes involved in the adaptive response to anaerobiosis, acid stress, and high osmolarity [137,138]. HU binds to double-stranded DNA with low affinity and without sequence specificity. However, it binds specifically and with high affinity to defined DNA structures [139,140,141]. Studies on the transcription of the *gal* operon of *E. coli* showed that its repression by GalR and HU requires a structure-specific HU binding [142]. In the case of *Streptococcus pneumoniae*, the SpnHU protein, which seems to be the only NAP encoded by this bacterium, was shown to be essential to maintaining the superhelicity of its chromosome [143]. Moreover, recent results suggest that the structure-specific HU binding mode would occur at limited bacterial chromosome sites and would regulate gene expression, most likely by promoting the formation of local higher-order DNA structures at or near promoters [144].

The Mga/AtxA family of global regulators includes Mga from *S. pyogenes*, AtxA from *B. anthracis*, Mga*Spn* from *S. pneumoniae*, and MafR from *Enterococcus faecalis*. These regulatory proteins act mainly as transcriptional activators. They have been implicated in bacterial virulence and several studies highlight their role in bacterial adaptation to specific host niches [145,146,147,148,149,150,151,152]. The three-dimensional structures of AtxA (PDB 4R6I; [153]), Mga*Spn* (PDB 5WAY; unpublished) [154], and MafR (PDB 3SQN; unpublished) have been solved. Bioinformatics analyses have shown that the members of the Mga/AtxA family have a similar organization of functional domains, including a helix-turn-helix DNA-binding domain within the N-terminal region followed by PTS regulation domains (known as PRDs) [146,150,153]. Investigations on the AtxA and Mga regulators support that their activity is modulated by PTS-mediated phosphorylation of histidine residues located within PRDs [155,156,157]. Regarding the protein-DNA recognition mechanism, it has been proposed that the regulators of the Mga/AtxA family have a preference for particular DNA structures rather than for specific nucleotide sequences [154,158]. This proposal is supported by several findings, such as (i) Mga*Spn* and MafR binding to linear double-stranded DNAs with little or no sequence specificity [154,158]; (ii) Mga*Spn* having a high affinity for a naturally occurring curved DNA and a preference for AT-rich DNA regions [154,159]; (iii) DNA sites recognized by MafR containing regions of potential bendability [151,158]; (iv) alignments of DNA regions recognized by Mga revealing low sequence identity [160]; and (v) the promoter regions of some AtxA target genes being intrinsically curved [161]. A recent study suggested that the pneumococcal PclR protein, which activates transcription of the *pclA* gene (collagen-like protein), is a potential member of the Mga/AtxA family [162]. PclA was shown to mediate pneumococcal adherence to human cells in vitro [163]. 

## 5. One Earth: Final Remarks and Future Perspectives

The *One Earth* concept was previously proposed by us to create a human conscience toward considering all biological entities as a single unit [14]. In its light, the presence of ARGs and their dispersal by HGT is considered a revenue of biodiversity and not a threat to the biosphere. Our understanding of biodiversity has also changed over the years and it counts on the resiliency and fitness of any given ecosystem rather than on the number of species occupying a given niche [164]. At present, entire ecosystems are being transformed, either by anthropogenic activities or as a consequence of climate change (also human-provoked). These changes severely affect the functionality of the ecosystems, and their self-recovery capacity will be impaired (https://wellcome.org/news/climate-change-antimicrobial-resistance; accessed on 1 August 2023). The consequence of the entire planet’s deterioration is a serious decay of its biodiversity and, ultimately, biodiversity-drained ecosystems will affect the climate.

Under the *One Earth* umbrella, we should reinterpret the AMR as a result of human intervention and selection of ‘superbugs’ due to biomedical, veterinary, and crop concerns. Research and development on infectious diseases presently require an ambitious re-design because many countries are being left behind. Infectious diseases are responsible for ~25% of deaths globally and these numbers seem to be increasing. However, if we aim to attain solutions, we should contemplate the global ecological importance of our participation in the prevalence of the high resistance achieved in recent years as well as the possible solutions [165]. Knowledge of the ecological role of antibiotic biosynthetic pathways and the antibiotic resistance developed by bacteria living in natural communities is of essence to implementing policies on how to deal with bacterial infections [166]. Consideration of antibiotics as war-like objects to fight bacterial infections has not benefitted our understanding of the bacterial world. On the contrary, we have envisaged bacterial communities as places where there is a continuous battle to win scarce resources. Different views, however, support the idea that polybacterial communities can cooperate through the employment of signals, like QS or phosphorylation relays [104]. Global solutions concerning the microbiome must be taken, and a set of them includes: (i) conservation; (ii) restoration; and (iii) biofilm management [167], taking into consideration that several misconceptions or even mistakes have been related to the microbiome research [168].

As **future perspectives**, we propose comprehending the bacterial world and its interactions with other living beings and explaining how bacterial populations behave and communicate in the different complex niches they inhabit. In addition, we can start understanding the relationships between the human species and its microbiomes, so that we can globally influence our environment. Then, we can begin to understand how to diminish bacterial virulence as an alternative strategy to cope with bacterial infections. Employment of antivirulence therapies that do not inhibit bacterial growth but diminish the synthesis of virulence factors would be the better way to cope with the present situation of AMR. Understanding the nichomes and their complex relationships requires knowledge of the conditions under which the global regulators are expressed. It will be this holistic *One Earth* concept that will help to find an equilibrium between the human and the bacterial worlds [169].

Our conclusion opts to cite verbatim the English writer Samuel Butler (“The Way of all Flesh”, 1864): “All our lives long… we are engaged in the process of accommodating our changed and unchanged selves to changed and unchanged surroundings; living is nothing else than this process of accommodation…”.

## Figures and Tables

**Table 1:** Antibiotics: targets and resistance ^a^.

Antibiotic Type: Examples	Targets	Resistance
β-Lactams: penicillins, cephalosporins	Enzymes involved in cell-wall synthesis	Breakage of the β-lactam ring by β-lactamases. Decreased uptake or increased efflux of the β-lactam. Changes in the active site of the target.
Cationic peptides: colistin, polymyxin B	Bacterial membrane	Lipopolysaccharide modifications. Efflux pumps. Capsular polysaccharide production.
Fluoroquinolones: ciprofloxacin	DNA gyrase and topoisomerase IV	Mutations in target enzymes.
Rifamycins: rifampin, rifabutin, rifapentine	RNA polymerase	Mutations in the RNA polymerase (β subunit). Drug inactivation. Impeded uptake of the drug.
Aminoglycosides: streptomycin, gentamicin	30S ribosomal subunit	Enzymatic modification of the drug. Enzymatic modification of the target site. Efflux pumps.
Tetracyclines: tetracycline, doxycycline, minocycline	30S ribosomal subunit	Efflux pumps. Ribosomal protection proteins. Enzymatic inactivation of tetracycline.
Phenicols: chloramphenicol, thiamphenicol, florfenicol	50S ribosomal subunit	Ribosomal mutations. Enzymatic modification (methylation) of the target site. Enzymatic inactivation (acetylation) of the drug. Efflux pumps.
Macrolides: erythromycin, azithromycin, clarithromycin	50S ribosomal subunit	Ribosomal modification by methylation or mutation. Efflux pumps. Drug inactivation.
Lincosamides: clindamycin	50S ribosomal subunit	Ribosomal modification by methylation or mutation. Efflux pumps. Drug inactivation.
Sulfonamides: sulfamethazine, sulfadiazine	Dihydropteroate synthase in the folic acid pathway	Mutations in the target enzyme. Sulfonamide resistance mediated by HGT (genes encoding variants of the target enzyme). Drug degradation.

^a^ Further information can be retrieved from the Comprehensive Antibiotic Resistance Database (https://card.mcmaster.ca/; accessed on 31 July 2023).

**Table 2:** Relevant bacterial MGEs.

Element	Main Features
Plasmids	DNA molecules that replicate autonomously.
Conjugative Plasmids Mobilizable Plasmids	Encode all elements needed for conjugation. Encode a relaxase and an origin of transfer. Need the conjugative machinery provided by ‘auxiliary’ plasmids.
Integrative and Conjugative Elements (ICEs)	Integrated elements that can excise, replicate, and transfer by conjugation. Previously termed ‘Conjugative Transposons’.
Integrative and Mobilizable Elements (IMEs)	Integrated elements that can excise and replicate but need conjugative machinery provided by ‘auxiliary’ elements for their transfer. Previously termed ‘Mobilizable Transposons’.
Insertion Sequences (ISs)	Integrated elements that encode only transposition machinery.
Prophages	Bacteriophage genomes totally or partially inserted into the host chromosome.
Phage satellites	Elements that exploit phages for transfer between bacteria and can encode ARGs.

## Data Availability

No new data were created or analysed in this study. Data sharing does not apply to this article.

## Abbreviations

AI, artificial intelligence; AMR, antimicrobial resistance; ARGs, antibiotic resistance genes; COINS, conjugation inhibitors; EPs, efflux pumps; HGT, horizontal gene transfer; H-NS, histone-like nucleoid structuring protein; ICEs, integrative and conjugative elements; IMEs, integrative and mobilizable elements; ISs, insertion sequences; MGEs, mobile genetic elements; NAPs, nucleoid-associated proteins; PRDs, PTS regulation domains; PTS, phosphoenolpyruvate:carbohydrate phosphotransferase system; QS, quorum sensors; TCSs, two-component signal transduction systems; T4SS, T4 secretion system.
